# Creation and use of organoids in biomedical research and healthcare: the bioethical and metabioethical issues

**DOI:** 10.1080/19336918.2021.1996749

**Published:** 2021-10-28

**Authors:** Henri-Corto Stoeklé, Achille Ivasilevitch, Geneviève Marignac, Christian Hervé

**Affiliations:** a Department of Ethics and Scientific Integrity, Foch Hospital, Suresnes, France; b Laboratory of Business Law and New Technologies (Dante) (UR4498), Paris-Saclay University (Uvsq), Montigny-Le-Bretonneux, France; c Ecole Nationale Vétérinaire d’Alfort, Maisons-Alfort, France; d University of Paris, Paris, France; e International Academy of Medical Ethics and Public Health, University of Paris, Paris, France; f Veterinary Academy of France, Paris, France

**Keywords:** Organoid, biomedical research, healthcare, bioethics, metabioethics

## Abstract

In the field of bioethics, scientific articles have already been published, and have highlighted relatively pluralist reflections concerning the creation and use of organoids. This plurality, rather than simply reflecting the complexity of the subject, may also be a consequence of the multiple theoretical and practical frameworks applied. Moreover, the creation and use of organoids in biomedical research and healthcare is probably in its infancy. This phenomenon is likely to increase in amplitude. Bioethics may be able to provide it with an effective and pertinent moral meaning, provided that a veritable metabioethical reflection is developed in parallel, that is, a reflection on bioethics itself, to provide scientists and clinicians with the best possible assistance in their everyday practice.

## Introduction

What began as an idea, not so long ago, is, little by little, becoming a reality: the creation and use of ‘organoids’^1^ in the frameworks of biomedical research and healthcare [1–6].

Organoids are ‘stem cell-derived or progenitor cell-derived 3D structures that, on much smaller scales, re-create important aspects of the 3D anatomy and multicellular repertoire of their physiological counterparts and that can recapitulate basic tissue-level functions.’ [7]. They are living, three-dimensional structures obtained from human cells cultured *in vitro*, or *in vivo*, in chimeric animals [1]. At least three types of cells are used: induced pluripotent stem cells (iPSCs), embryonic stem cells (ESCs) and adult stem cells^2^ [2]. Adult stem cells can be either normal or pathological ones as tumoroids, in particular, are more and more generated. This new biotechnological technique makes it possible to obtain a bioartificial organ or part of an organ, such as a kidney, a liver, or even a brain, in which case it is known as a ‘cerebroid’ [3,8]. The history of organoids as a research model dates back at least to the 1980s [1,3,8,9], if not even earlier [10]. However, their history in terms of real or potential use in clinical applications, particularly in the framework of ‘personalized medicine’, ^3^ ‘regenerative medicine’^4^ and ‘reproductive medicine’, ^5^ seems to be a bit more recent [1,11]. Regardless, scientific and clinical interest in organoids is currently increasing. Indeed, using human cerebroids, researchers were recently able to provide concrete evidence of neurodevelopmental changes associated with the microencephaly caused by Zika virus [12–15], and, in 2017, the scientific journal Nature Methods honored organoids with the title of ‘method of the year’ [12,16].

In bioethics, a certain number of scientific articles have already been published, highlighting a relatively pluralist reflection on the creation and use of organoids in the frameworks of biomedical research and healthcare [1,4,5,11,12,17–27]. Four major bioethical problems and issues – that is, moral (and/or legal) tensions, the resolution of which would have an impact on the reasons for creating and using organoids, or the ways in which this is done, for example – emerge very clearly: biobanks and informed consent; clinical trials and the benefit/risk ratio; the moral status and level of consciousness and/or sensitivity of organoids; and alternative methods and animal welfare [28]. Nevertheless, on reading these articles, it becomes clear that certain publications and authors focus preferentially on one problem, and on specific bioethical issues, such as the moral status and level of consciousness and sensitivity of cerebroids or ‘gastruloids’, the term for bioartificial embryos [10,12,18–20,23,27,29].

We think that these choices were probably not made by chance, and that they were influenced by various factors. Thus, implicit in this bioethical reflection on organoids, there is a veritable ‘metabioethical’ reflection – that is, a reflection on bioethics itself, at both the theoretical and practical levels – that needs to be developed, to improve our understanding of the rationale and reasons pushing some bioethicists or bioethics researchers to focus more on a particular problem than on others, and to deal with issues in a particular fashion. In the framework of this article, we will limit ourselves to a specific geographic and/or cultural field: the ‘West’^6^ (Europe, North America, etc.), partly because most of the studies on bioethics published to date that we were able to identify seem to come from this geographic and cultural zone, and partly because our own field of competence and knowledge in the matter is also limited to this zone.

The objective here is to identify possible metabioethical issues from the bioethical issues identified in the scientific literature on bioethics relating to the creation and use of organoids in biomedical research and healthcare, and to try to envisage possible solutions.

## Bioethical issues

**Figure 1.**
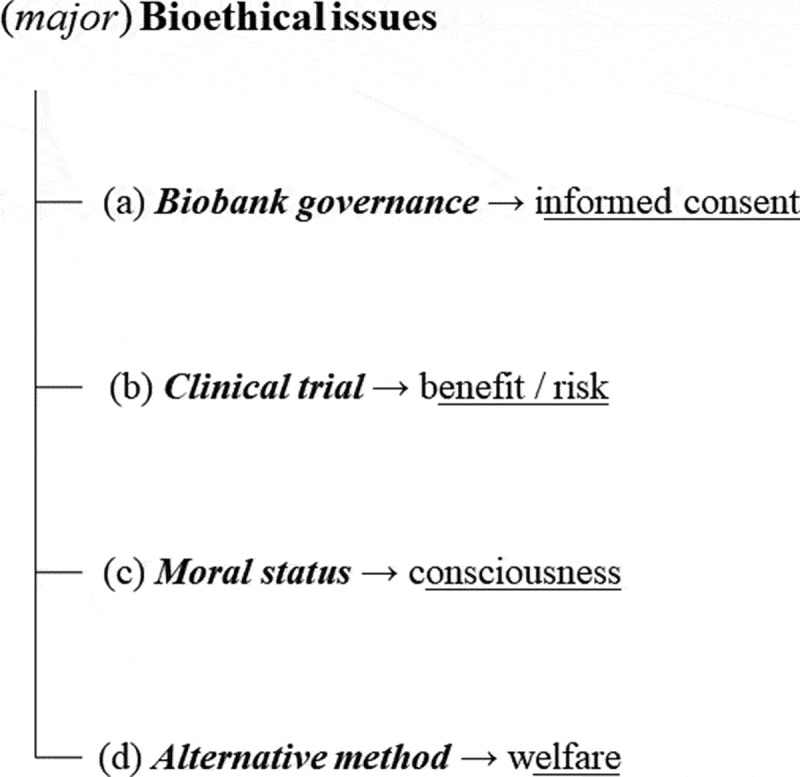
Diagram of the bioethical issues associated with the creation and use of organoids and gastruloids in biomedical research and healthcare

The creation and use of organoids in the framework of biomedical research generally implies the prior existence of biobanks storing various samples of human biological materials (e.g. organs, tissues or cells) collected for research purposes [1,30], or in the framework of healthcare, with subsequent repurposing for research use [1]. These are the biological samples used to produce the IPSCs, ESCs or adult stem cells required for the creation of an organoid, regardless of its type (liver, kidneys, etc.). In the scientific literature, the means and modes of biobank governance are starting to emerge as a first important bioethics issue concerning organoids (Figure 1(a)) [1,26,30]. A number of issues have been raised, including the type of consent used in the first place, or the choice between so-called ‘express’ or ‘explicit’ informed consent^7^ (*opt-in*^8^) and informed consent described as ‘presumed’^9^ or ‘implicit’^10^ (*opt-out*^11^) (Figure 1(a)) [1,27,31–34]. The key issue is knowing the level of decision-making to be shared with the donors of the biological samples from which the organoids will be created for research use. In this context, some people favor the use of implicit consent, or of explicit ‘broad’ consent,^12^ with donors playing a passive and anonymous role by consenting to the creation of organoids from their biological samples for various studies authorized by the biobank managers, according to the laws and/or regulations in force in the country in which the samples are collected and the research is performed [1].

Conversely, others prefer explicit ‘dynamic’ consent,^13^ which enables the donor to play a relatively active role. For this to be possible and operational, computer platforms and uses of information and communication technology (computers, computer tablets, smartphones, etc.) must be developed to facilitate dynamic, two-way exchanges of data and information between the donors and users of the biobank concerning specific research, for which consent is given [1,35,36]. One important feature of such consent is that these exchanges can change over space and time. Furthermore, let’s also remember that some countries, just like France,^14^ or United Kingdom, as well as journals, impose the tracing of the different human-derived cell lines [37]. These features highlight the interest of developing organoids preferentially from IPSCs or from adult stem cells rather than from embryonic stem cells, for which there are, in reality, two donors (the two parents), making it necessary to obtain two consents [27]. What’s more, these donors do not really speak in their own name, but on behalf of a ‘potential’ embryo – although this term could be discussed –, which renders the underlying bioethical problem even more complex, and has implications for the associated operational management.

Interestingly, organoids are generally subject to the same bioethical issues as personalized medicine in general: informed consent, ‘fortuitous’ discoveries^15^ and ‘literacy’.^16^ However, the legal ownership of the organoid itself – rather than just health data, particularly those of a genetic nature, or biological samples – and of any patents resulting from it [1,36,38–40] is also a key issue. But the link between organoids, personalized medicine and bioethics does not end there. Organoids will also be created for the testing of new therapeutic molecules, replacing animals and/or humans in this process [1]. In the case of cancers, it will be possible to create ‘tumoroids’, ^17^ tumor organoids, from the cancerous cells of a specific patient, for *in vitro* studies, or even *in vivo* studies in chimeric animals, of the specific response of the cancer patient to a particular treatment, particularly for experimental treatments, regardless of the affected organ (liver, pancreas, brain, etc.) [1,41]. Similarly, organoids could also be used to test certain ‘genome-editing’ techniques^18^ [42,43].

However, bioethical problems are not limited to personalized medicine. In regenerative medicine, organoids will not be used for testing medicines. Instead, they will themselves be the medicine, in the form of a graft [1,44–53]. This should make it possible to resolve the almost systematic shortage of organs available for transplantation, and to overcome the need for anti-rejection treatments, which are generally required for the rest of the patient’s lifetime. This will be possible because, in the future, organoids will be created from stem cells selected from a biobank of pluripotent stem cell lineages to be fully compatible with the recipient. For both personalized and regenerative medicine, the other key problem is that of clinical trials, and the bioethical issue of the benefit/risk balance for a particular patient in these two very precise situations (Figure 1(b)): on the one hand, administering an experimental treatment that has never been tested in preclinical research on animals, but only on organoids, or tumoroids, as some could imagine, even if it is difficult, or impossible, to do so, at least for the moment; and, on the other, transplanting, for the first time, an organ in a purely experimental framework [1,44–46,48,49]. Is it an insurmountable obstacle? It depends. Such an approach should be carefully framed, as it has been the case for the first natural or artificial organ transplantation, of course taking into account changes in the societal context on these sensitive ethical points.

In the very specific case of cerebroids and gastruloids, the moral status of the organoid, as a ‘patient’ or ‘moral agent’ [54], and its level of consciousness and sensitivity appear, as indicated in the introduction, to be a problem in which bioethical issues come to the fore (Figure 1(c)) [1,12,19,23,24]. We can distinguish here a polarization of the debate relatively similar to that concerning the creation and use of embryos in the framework of research [1,2,10,12]. Indeed, the association of the notion of a ‘human person’ or a ‘potential human person’ with the organoid, due to the source of the cells and the possibility of a form of consciousness, renders certain people highly reticent, or even outright opposed to the development of organoids, according to their underlying philosophical and/or religious principles. Conversely, others are relatively favorable to the creation and use of organoids, subject to certain provisos, including the existence of a real or potential medical need for humans, limitation of the suffering inflicted on the organoid and the obtainment of free, informed consent from the donor or donors. Once these aspects have been outlined, the debate appears much more complex, and non-binary.

Legally, the creation of organoids – including cerebroids and gastruloids – in research seems to be widely authorized in most Western countries, or at least, as in France, the technique does not seem to be strictly prohibited [1,10,54]. There are probably multiple, diverse reasons for this, including the very recent nature of the techniques concerned. However, it is also possibly because organoids, including cerebroids and gastruloids, correspond to alternative research methods of considerable interest as a possible replacement for the various models habitually used, which are (bio)ethically problematic. However, if we take the example of gastruloids, these organoids appear to be more of a complementary solution than an alternative to research on human embryos. Real human embryos will still be required, to check the results obtained with gastruloids, particularly in clinical research or scientific research for medical purposes [1]. In any case, animal welfare, or the idea of combating animal suffering thanks to the use of organoids as an alternative research approach in research, is an important issue to be considered here (Figure 1(d)).

The question of the use and/or killing of animals for scientific and/or clinical purposes is a recurrent legal, moral, ethical and bioethical debate [55,56] every bit as complex as that of the creation and/or use of human embryos for the same purposes. Furthermore, it cannot be reduced to a binary opposition of ‘for’ and ‘against’. For example, some of its opponents are not willing to accept any use, and even less any killing of animals, even if the research involved can have real major medical benefits for humans. Others have a less entrenched position and wish to ensure continual decreases in the number of animals used or killed during research, at least in the short and medium term [1,57,58]. The social cartography of opponents is also probably far from simple. Their stance has led to many efforts being made, notably in France, to decrease the proportion of animals used or killed, to provide arguments for the use and killing of animals, through a requirement for approval from an animal research ethics committee^19^ for all projects involving animals, and even to justify the use of animals, because retrospective evaluations are sometimes requested [59]. The creation, albeit recent, of animal welfare structures at each research institute in France, was designed to take the needs of the animals into account more effectively. Finally, methods for replacing animals are now part of the initial and ongoing professional training of researchers and animal house technicians [59].

For many, organoids appear to be a means of reducing the proportion of animals used further, and possibly even of overcoming the need for animal models altogether. However, this ‘generation’ of organoids is still subject to significant scientific and technical limitations, such as an absence of blood vessels, nerves or an immune system, making it difficult, if not impossible, to extrapolate the results obtained to humans, particularly in the domain of health [1,2]. For this reason, certain organoids are implanted in animals. The moral status of the chimera obtained in this manner is possibly even more problematic than that of the organoid itself [10]. As a means of resolving this problem, some people envisage the development of robots or computers capable of simulating the bloodstream or the nervous system [2]. However, at this stage, such as the use of human embryos in biomedical research, organoids are seen more as a complementary solution than as an alternative to animal experimentation [1].

Perhaps we can now see this plurality of bioethical reflection on the subject of organoids, as mentioned in the introduction, more clearly. This plurality, rather than simply reflecting the complexity of the subject, may be a consequence of the history of bioethics, combined with theoretical and practical frameworks that are just as plural.

## Metabioethical issues

The precise origin and field of ‘bioethics’ are far from evident in themselves [60–68]. From a temporal standpoint, bioethics can be considered to have come into existence during the second half of the 20^th^ century. In terms of geography, it arose in the West, and, more precisely, in the United States. Indeed, the American bioethicist and biochemist Van Rensselaer Potter, Professor of Oncology at the University of Winsconsin, published a scientific article in 1970, followed by a book in 1971 that are often considered to be the first works fleshing out this concept, even though others seem to have used this concept before him [60,69–71]. For Potter, bioethics was a solution to what he saw as the broken link between biology and ethics, not, for him, a means of imposing a moral framework that should never be transgressed, but rather endowing biology with an ethical objective, beyond the sole objective of producing scientific or medical knowledge: improvement of the quality of life and survival of each and every member of society, largely taking into account the environment (biodiversity, natural resources, etc.). Continuing along these lines, we can also say that scientific and technical innovations, particularly those that are ethically problematic for society and its individuals, should not be judged on moral grounds as a function of theoretical values and standards. Instead, they should be studied scientifically, from an ethical, but practical standpoint. This ‘global bioethics’ can thus be distinguished from ‘medical bioethics’, which emerged shortly after the initial work of Potter, at the initiative, *a priori*, of the American bioethicist and obstetrician André Hellegers, Professor at the University of Georgetown.

It is the medical aspect of bioethics that is most widely practiced today in the Western world, to the point that many see the term ‘bioethics’ as restricted to the medical field [62,65,72]. The practice of this medical form of bioethics was first developed at the Hasting Center,^20^ and was rapidly adopted thereafter at the Kennedy Institute of Ethics^21^ in the United States. It aims to establish a normative framework that is more or less restrictive, to be applied to new practices and/or techniques emerging in the field of medicine. The most famous advocates of this medical bioethics (also known as ‘biomedical ethics’) were probably the American bioethicists and philosophers Tom Beauchamp and James Childress [73]. The Canadian bioethicists and theologians David Roy and Guy Durand, together with Hubert Doucet [74], and the Belgian bioethicist and philosopher Gilbert Hottois [68], worked to promote a dynamic form of medical bioethics, envisaging ethical problems on a case-by-case basis, rather than applying so-called ‘general’ principles, as in the school established by Hellegers and in the principlism of Beauchamp and Childress [75,76].

It is, thus, clear that the theoretical framework of bioethics is far from homogeneous. It may even be considered excessively heterogeneous, particularly if we establish no semantic and conceptual distinction between bioethics and ‘philosophical ethics’, ^22^ also known as ‘moral philosophy’, ^23^ or even ‘religious ethics’^24^ [75,77,78]. Three genres can currently be distinguished in philosophical ethics: ‘normative ethics’, ^25^ ‘applied ethics’, ^26^ which can also be included in normative ethics, and ‘metaethics’^27^ [79]. The first of these categories covers various ‘ethical theories’^28^ that can be used to reach a moral judgment about any action, whatever its nature. The second aims to deduce or to apply general and theoretical principles to specific practical situations. The last category analyzes the theoretical and practical bases of this philosophical ethics.

Multiple ethical theories are used in the field of normative ethics, but they can all be grouped together into at least three large families: ‘deontological ethics’, ^29^ ‘consequentialism ethics’, ^30^ which is the most used of the ‘teleological ethics’, ^31^ and, finally, ‘virtue ethics’, ^32^ which differs from the other two in different ways, according to the authors concerned [28]. For example, English-speaking bioethicists currently display a propensity to interpret this form of ethics in an almost teleological manner [79,80]. Bioethicists such as Roy, Durand and Potter, to name but a few, established a semantic and conceptual distinction, of varying degrees of precision, between bioethics and philosophical ethics, and, indeed, religious ethics. By contrast, most of the bioethicists currently active do not seem to make this distinction, regardless of whether or not they are philosophers. They rarely state which train of thought from philosophical ethics they are using. For example, two of the ethical principles proposed by Beauchamp and Childress are utilitarian, and two are more deontological, but this choice is neither explicitly stated nor clearly analyzed. Some authors go so far as to make the use of these principles a branch of ethics, whereas it is actually an aggregation of different schools of philosophical ethics, borrowing terms from healthcare and politics for application in the field by healthcare professionals.

Similarly, as concerns organoids and the bioethics research studies we were able to identify, the problems considered relates primarily to medical bioethics, generally confounded with philosophical ethics, in its normative and applied form. The approaches used also appear to be highly heterogeneous. Different theories are used, and sometimes mixed, with various degrees of explicitness, as shown, indirectly, in the review article published by Bredenoord *et al*. in Science in 2017, widely cited in the first part of this work [1,28]. We can thus distinguish ‘kantianism’^33^ or the establishment of a moral duty to organoids if they are conscious; ‘utilitarianism’, ^34^ in which a moral duty to organoids is established if they suffer; ‘principlism’, ^35^ in which the four major principles of autonomy, benevolence, non-malevolence and justice toward the donor must be respected; the ‘precautionary principle’, ^36^ in which the worst-case scenarios relating to the creation and use of organoids are considered, and ‘the ethics of discussion’, ^37^ which involves collectively reflecting and deliberating on the choices considered most desirable, for society in particular. Probably, other theories may be identifiable. In any case, it is perhaps easier to understand now the idea that the plurality of bioethical reflections on organoids is not solely linked to the intrinsic complexity of this biotechnology. It is also largely influenced by the theoretical framework in which the bioethicists responsible for this reflection operate.

**Figure 2.**
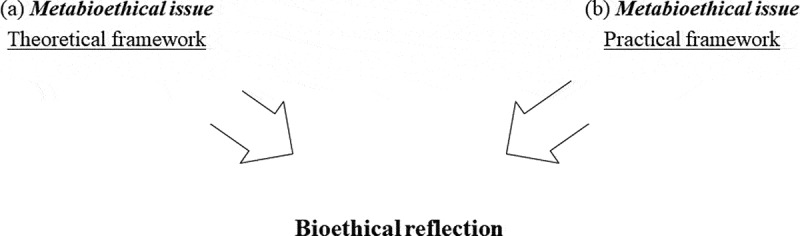
Diagram of the metabioethical issues or factors that could influence bioethical reflections on the creation and use of organoids in biomedical research and healthcare

The theoretical framework is, thus, a metabioethical issue in itself, as we can perceive an ‘epistemological’^38^ tension concerning the orientation of the bioethical reflection, and an even greater tension concerning the reasons and/or ways of creating and using organoids, whether in biomedical research or healthcare (Figure 2(a)). Another important point is that this heterogeneity is visible at the global scale, and possibly most markedly in the English-speaking world and in areas culturally influenced by this world. In certain regions of the world, especially in France, reflections appear more homogeneous, and considerations of this type concerning organoids are more often moral than ethical.

For the French philosopher Paul Ricoeur [81], and for other philosophers, such as the German Jürgen Habermas, morality is a sort of highly contemporary translation of deontological ethics, and the ethics concerned is teleological ethics. Morality relates more to duty, and to an ethics of ends and of meaning [82]. It could also be said that, in the face of an action, morality is the application of a normative framework, a collection of values (or ‘should-bes’) and standards (or ‘should-dos’). It corresponds to ‘how’ this should be or that should be done in the face of this action. Ethics is more a criticism of this framework: it answers the question ‘why’ this should be or that should be done, particularly if the action and the framework are out of step with each other [56,83,84]. Indeed, we see that the creation and use of organoids is more subject to prescriptions than to deliberation, as shown above.

In addition, most scientific publications on organoids in the field of bioethics, in the broad sense of the term, originate principally from the English-speaking world, also in the broad sense of the term, such as the Department of Bioethics at Case Western Reserve University School of Medicine in Cleveland in United-States,^39^ the Biomedical Ethics Research Groups at Murdoch Children’s Research Institute in Melbourne in Australia,^40^ or the Department of Medical Humanities at University Medical Center Utrecht in the Netherlands^41^; French publications are rare [2,85]. This subject has, to date, principally been dealt with through four reports,^42^, ^43^, ^44^ including one published in 2020 by the ethics committee of INSERM (the French National Institute for Health and Medical Research)^45^ entitled ‘Research on organoids: the ethical issues’ [10]. One of the reasons for this lack of French publications may be the practical framework of bioethics in France, where bioethics is mostly seen as being ‘institutional’, ^46^ rather than ‘academic’, ^47^ in the sense of a university discipline [75,76]. That does not mean that there are no bioethics research structures (laboratories, teams) in universities or elsewhere in France; indeed, the contrary is true,^48^, ^49^ .^50^ Instead, political decision-makers in France, for reasons that are probably cultural, relatively complex and long-standing, decided at some point that bioethics would be practiced essentially through ethics committees (*Comité consultatif national d’éthique* (CCNE),^51^ *Comité d’éthique de l’Inserm, Comité d’éthique du Center national de la recherche scientifique* (CNRS) (COMETS),^52^ etc.), and through ‘ethical reflection spaces’ (*Espaces de réflexion éthique*, ERER),^53^ which mostly produce reports or expert opinions, rather than scientific articles, destined for their overseeing institutions (INSERM, CNRS, etc.), political decision-makers and/or public agencies (*Agence de biomédecine*,^54^ *Commission nationale l’informatique et des libertés* (CNIL),^55^ etc.). In this framework, discussions focus on the response to questions raised by society or by a particular organization, rather than on more fundamental issues concerning the status of organoids, with explicit reference to the philosophical and conceptual frameworks used: the very opposite of the process followed by researchers in ethics. Furthermore, on these committees, bioethicists as professionals (as researchers or lecturer-researchers) and bioethics as a new academic discipline in its own right, original in the sense that it is interdisciplinary in nature, are accorded little if any recognition, contrasting strongly with the situation in the United States and Canada, for example. It is probably not by chance that a certain number of scientific articles cited here come from research teams, laboratories or departments of Universities, Institutes, in countries where bioethics tends to be practiced as an academic discipline.

The relative homogeneity of bioethical reflections on organoids in France may be a consequence of this practical framework. Indeed, institutional publications on bioethics in France tend to make considerable use of deontological ethics in the argumentation of their proposals, and the ethics of discussion is widely used to achieve this end [76]. In addition, over the last two decades or so, interest in the precautionary principle – an ethical theory derived from consequentialism ethics^56^ – has greatly increased, even though the way in which this principle is applied in France is based more on deontological ethics. The replacement of the term initially used by Hans Jonas (responsibility) by ‘precaution’ in the law is very revealing. For example, deontological ethics – or, in a certain sense, morality – and particularly the theory proposed by Kant, are identifiable in the report published by the ethics committee of INSERM, in which the bioethical problem of the moral status of organoids according to whether or not they are conscious is favored, particularly for cerebroids, over the issues of their use and development, which receive little attention [10].

Thus, the practical framework of bioethics, that is, the way in which bioethical discussions about practical cases are conducted, is not devoid of metabioethics issues. Effectively, there is a tension, probably cultural or even political in nature, in the choice between academic and institutional bioethics [76]. These choices affect the orientation of bioethical reflections on these bioartificial organs and, thus, again, on the reasons and ways of creating and using organoids in the framework of research and/or healthcare (Figure 2(b)).

## Conclusion and perspectives

As for metaethics and philosophical ethics, we think it would be useful to develop metabioethics in bioethics, to render explicit the framework of this reflection, and, thus, to increase pertinence and avoid omissions in reflections about the creation and use of organoids in research and healthcare. Based on this example, all other bioethical topics, such as genetics, cloning, genetically modified organisms, chimeras, medically assisted procreation, surrogacy and artificial intelligence, could be approached with this methodological explanation. Indeed, in France, this reflection makes it possible to highlight the reasons that might lead to the problem of moral status and the issue of consciousness being favored [10]. Epistemological and cultural aspects are far from being subsidiary issues in bioethics. It would also be useful to pursue this bioethical and metabioethical reflection on the creation and use of organoids elsewhere than in the West. The diversity of cultures does not exclude the existence of common concerns regarding organoids.

By contrast to this chain of thought, most of the research work published at the moment is theoretical in nature [86]. It will be important to perform more empirical bioethical research, to draw on the questions asked of ethics committees and the experiences of healthcare professionals and researchers to develop a base of theoretical work. We would go even further, by saying that a more pragmatic approach to bioethical reflection could be appropriate. In other words, the bioethical issues and the need to resolve them according to practical and anthropological objectives could be inferred from experience, to improve quality of life and individual and collective survival, for example [28]. This might be more appropriate than simultaneously trying to deduce and resolve these issues by reason or on the basis of convictions, as a function of rules that often diverge, such as certain religious, philosophical, political and even scientific dogmas [87]. In summary, we propose a return to a bioethics that is more global than medical [72]. The question would then no longer be solely to determine whether the creation and use of organoids transgress a normative framework and whether or not we should enforce this framework. Instead, it would be more a case of seeking to understand why and how questions emerge, of envisaging the ways in which this biotechnology could potentially improve the quality of life and survival of humanity, by acting case-by-case, and taking the cultural specificities of countries and individuals into account. Indeed, different cultures sometimes have radically different relationships to the human body, spirit and nature. Bioethicists needs to take these differences into account.

In this context, a professionalization of bioethics also appears necessary. Interdisciplinarity is not enough in itself. We need professionals in bioethics, that is, researchers and researcher-lecturers with an appropriate level of knowledge and skill in the matter, either profoundly transdisciplinary (science, medicine, philosophy, law, theology, etc.) or in a position to work full-time on these complex subjects. Bioethical reflections are becoming increasingly essential for the satisfactory, or optimal development of biotechnologies in our societies. And these ‘bioethicists’ should no longer be solely, or principally, philosophers, jurists or theologists by training. They could, or rather should be doctors, pharmacists, nurses, scientists, engineers or veterinary surgeons. In other words, it is the teaching of bioethics and research in bioethics that should be developed and diversified, to ensure the effective direct assistance, on a daily basis, of scientists and clinicians.

To this we must add the teaching of and research into ethics and scientific integrity. The development of a more pragmatic and academic bioethics, promoting more empirical and ‘inductive’^57^ research based on scientific facts and practices rather than the simple ‘deduction’^58^ of theoretical values and/or standards, requires these practices and factors to exist in a characterized moral framework that includes empirical and pragmatic approaches [28]. The construction and (necessary) evolution of this moral framework should be based on the direct observation of scientific practices and/or techniques on the one hand, and evaluation of the ethical efficiency of this framework as a function of the effective improvement in knowledge production and its positive impact on society and its individuals on the other. This role should preferentially be taken up by scientists themselves, trained in concepts and methods – largely inspired by those existing in the human and social sciences – enabling them to perform this work, which, like bioethics, should, in the long term, become a true academic discipline covering a whole set of teaching and fields of research of its own.

In conclusion the creation and use of organoids in biomedical research and healthcare have only just begun. This phenomenon is likely to increase in magnitude in the coming decades. Bioethics could provide it with an effective and pertinent moral sense, provided that it is accompanied by the parallel development of a real reflection in metabioethics, to provide scientists and clinicians with the best possible assistance in their everyday practices.

## Notes

1. https://www.cancer.gov/publications/dictionaries/cancer-terms/def/organoid.

2. https://www.mayoclinic.org/tests-procedures/bone-marrow-transplant/in-depth/stem-cells/art-20048117.

3. https://www.genome.gov/genetics-glossary/Personalized-Medicine.

4. https://www.mayo.edu/research/centers-programs/center-regenerative-medicine/about/about-regenerative-medicine.

5. https://www.cancer.gov/publications/dictionaries/cancer-terms/def/reproductive-medicine?redirect=true.

6. https://www.sciencedaily.com/terms/western_world.htm.

7. https://www.hrb.ie/funding/gdpr-guidance-for-researchers/gdpr-and-health-research/consent/explicit-consent.

8. https://dictionary.cambridge.org/fr/dictionnaire/anglais/opt-in.

9. https://dictionary.cambridge.org/fr/dictionnaire/anglais/presumed-consent.

10. https://www.law.cornell.edu/wex/implied_consent.

11. https://dictionary.cambridge.org/fr/dictionnaire/anglais/opt-out.

12. https://www.hrb.ie/funding/gdpr-guidance-for-researchers/gdpr-and-health-research/consent/broad-consent.

13. https://www.hra.nhs.uk/about-us/news-updates/what-dynamic-consent.

14. https://www.service-public.fr/professionnels-entreprises/vosdroits/R17957.

15. https://www.health.harvard.edu/staying-healthy/what-to-do-about-incidental-findings.

16. https://dictionary.cambridge.org/fr/dictionnaire/anglais/literacy.

17. https://www.thermofisher.com/fr/fr/home/life-science/cancer-research/solid-tumor-research/organoids-sperioids-3d-cell-cultures.html.

18. https://www.inserm.fr/en/health-information/health-and-research-from-z/genome-editing.

19. https://www.inserm.fr/professionnels-recherche/recherche-pre-clinique/comites-ethique-en-experimentation-animale.

20. https://www.thehastingscenter.org.

21. https://kennedyinstitute.georgetown.edu.

22. https://campuspress.yale.edu/stephendarwall/philosophical-ethics.

23. https://plato.stanford.edu/entries/morality-definition.

24. https://www.wiley.com/en-us/Religious+Ethics%3A+Meaning+and+Method-p-9781405198578.

25. https://www.britannica.com/topic/normative-ethics.

26. https://www.britannica.com/topic/ethics-philosophy/Applied-ethics.

27. https://plato.stanford.edu/entries/metaethics.

28. https://www.sciencedirect.com/topics/computer-science/ethical-theory.

29. https://plato.stanford.edu/entries/ethics-deontological.

30. https://plato.stanford.edu/entries/consequentialism.

31. https://www.britannica.com/topic/teleological-ethics.

32. https://plato.stanford.edu/entries/ethics-virtue.

33. https://www.britannica.com/topic/Kantianism.

34. https://www.britannica.com/topic/utilitarianism-philosophy.

35. https://www.sciencedirect.com/topics/psychology/principlism.

36. https://www.europarl.europa.eu/thinktank/en/document.html?reference=EPRS_IDA(2015)573876.

37. http://bibliotecadigital.fgv.br/dspace;/handle/10438/19481.

38. https://www.britannica.com/topic/epistemology.

39. https://case.edu/medicine/bioethics.

40. https://www.mcri.edu.au/biomedicalethics.

41. https://juliuscentrum.umcutrecht.nl/nl/medical-humanities.

42. https://www.etatsgenerauxdelabioethique.fr.

43. https://www.ccne-ethique.fr/fr/actualites/lavis-129-contribution-du-ccne-la-revision-de-la-loi-de-bioethique-est-en-ligne.

44. http://www.senat.fr/dossier-legislatif/pjl19-063.html#timeline-15.

45. https://www.inserm.fr/gouvernance-organisation/comite-ethique.

46. https://dictionary.cambridge.org/fr/dictionnaire/anglais/institutional.

47. https://dictionary.cambridge.org/fr/dictionnaire/anglais/academic.

48. https://lillethics.com.

49. https://www.u1027.inserm.fr/equipe-bioethics.

50. https://cesp.inserm.fr/fr/equipe/recherches-en-%C3%A9thique-et-en-%C3%A9pist%C3%A9mologie.

51. https://www.ccne-ethique.fr.

52. https://comite-ethique.cnrs.fr.

53. https://solidarites-sante.gouv.fr/systeme-de-sante-et-medico-social/ethique-et-deontologie/article/qu-est-ce-qu-un-espace-de-reflexion-ethique-regional-erer.

54. https://www.agence-biomedecine.fr.

55. https://www.cnil.fr.

56. https://www.vie-publique.fr/rapport/26392-le-principe-de-precaution-rapport-au-premier-ministre.

57. https://plato.stanford.edu/entries/logic-inductive.

58. https://www.sciencedirect.com/topics/mathematics/deductive-approach.
